# Early fine motor impairment and behavioral dysfunction in (Thy‐1)‐h[A30P] alpha‐synuclein mice

**DOI:** 10.1002/brb3.915

**Published:** 2018-02-04

**Authors:** Sara Ekmark‐Lewén, Veronica Lindström, Astrid Gumucio, Elisabeth Ihse, Anish Behere, Philipp J. Kahle, Eva Nordström, Maria Eriksson, Anna Erlandsson, Joakim Bergström, Martin Ingelsson

**Affiliations:** ^1^ Department of Public Health and Caring Sciences Uppsala University Uppsala Sweden; ^2^ Department of Neurodegeneration Hertie Institute for Clinical Brain Research and German Center for Neurodegenerative Diseases Tübingen Germany; ^3^ BioArctic AB Stockholm Sweden

**Keywords:** A30P mutation, alpha‐synuclein, behavioral outcome, challenging beam test, dementia with Lewy bodies, *Helicobacter pylori*, multivariate concentric square field test, Parkinson′s disease, transgenic mice, tyrosine hydroxylase

## Abstract

**Introduction:**

Intraneuronal inclusions of alpha‐synuclein are commonly found in the brain of patients with Parkinson's disease and other α‐synucleinopathies. The correlation between alpha‐synuclein pathology and symptoms has been studied in various animal models. In (Thy‐1)‐h[A30P] alpha‐synuclein transgenic mice, behavioral and motor abnormalities were reported from 12 and 15 months, respectively. The aim of this study was to investigate whether these mice also display symptoms at earlier time points.

**Methods:**

We analyzed gait deficits, locomotion, and behavioral profiles in (Thy‐1)‐h[A30P] alpha‐synuclein and control mice at 2, 8, and 11 months of age. In addition, inflammatory markers, levels of alpha‐synuclein oligomers, and tyrosine hydroxylase reactivity were studied.

**Results:**

Already at 2 months of age, transgenic mice displayed fine motor impairments in the challenging beam test that progressively increased up to 11 months of age. At 8 months, transgenic mice showed a decreased general activity with increased risk‐taking behavior in the multivariate concentric square field test. Neuropathological analyses of 8‐ and 11‐month‐old mice revealed accumulation of oligomeric alpha‐synuclein in neuronal cell bodies. In addition, a decreased presence of tyrosine hydroxylase suggests a dysregulation of the dopaminergic system in the transgenic mice, which in turn may explain some of the motor impairments observed in this mouse model.

**Conclusions:**

Taken together, our results show that the (Thy‐1)‐h[A30P] alpha‐synuclein transgenic mouse model displays early Parkinson's disease‐related symptoms with a concomitant downregulation of the dopaminergic system. Thus, this should be an appropriate model to study early phenotypes of alpha‐synucleinopathies.

## 1 INTRODUCTION

Parkinson's disease (PD) and dementia with Lewy bodies (DLB) are two of the most common neurodegenerative disorders. The PD and DLB brains are characterized by a progressive degeneration of neurons in both the nigrostriatal system and various cortical areas (Hughes, Daniel, Kilford, & Lees, 1992).

The motor symptoms in PD/DLB mainly result from the loss of striatal dopamine (DA), due to neurodegeneration of dopaminergic cells in substantia nigra (SN) (Bernheimer, Birkmayer, Hornykiewicz, Jellinger, & Seitelberger, 1973; Fahn, Libsch, & Cutler, 1971; Hirsch, Graybiel, & Agid, 1988). In addition, nonmotor symptoms are common and include anxiety, sleep disturbances, olfactory dysfunction, cardiac sympathetic denervation, and loss of cognitive functions (Aarsland et al., 1999; Chaudhuri, Healy, & Schapira, 2006).

Patients with both PD and DLB display Lewy body brain pathology, mainly composed of aggregated insoluble fibrillar forms of the presynaptic protein alpha‐synuclein (α‐syn) (Spillantini et al., 1997). The formation of such aggregates occurs via a number of steps in which various prefibrillar (oligomeric/protofibrillar) species are formed. These intermediate aggregates are believed to have toxic properties; causing disruption of cellular membranes (Danzer et al., 2007; Winner et al., 2011), mitochondrial toxicity (Di Maio et al., 2016), and inflammatory reactions (Wilms et al., 2009; Zhang et al., 2005). In addition to sporadic disease, mutations and/or multiplications in the α‐syn gene can lead to familial forms of PD and DLB, supporting the pathogenic significance of α‐syn (Lee & Trojanowski, 2006). Among the various α‐syn genetic variants, the A30P mutation causes an aggressive and early‐onset form of PD (Kruger et al., 1998).

Different environmental toxins, such as certain pesticides, have previously been indicated as external risk factors for PD (reviewed in (Goldman, 2014)). Moreover, observations of α‐syn pathology in the PD colon mucosa suggest retrograde spreading of pathology to the brain as a possible disease mediator (reviewed in (Lee et al., 2017)). Thus, the gut microbiota could possibly influence the brain pathology (reviewed in (Mulak & Bonaz, 2015)). Among the different microorganisms in the gut, *Helicobacter pylori* (*H. pylori*) could then be a particularly relevant species in this context, as PD has been associated with an increased prevalence of gastric ulcers (reviewed in (Alvarez‐Arellano & Maldonado‐Bernal, 2014)).

In recent years, transgenic (tg) mouse models for PD and other α‐synucleinopathies have become a valuable tool to study disease mechanisms and evaluate potential treatments. In tg mice overexpressing human A30P α‐syn under the Thy‐1 promotor ((Thy‐1)‐h[A30P] α‐syn tg mice), aggregated α‐syn accumulates in neuronal cell bodies and neurites (Kahle et al., 2000). Previously, these mice have been shown to display motor dysfunction, including end stage paralysis (Neumann et al., 2002), impaired coordination of movements, and cognitive impairment (Freichel et al., 2007). The cognitive dysfunction was found to correlate with the α‐syn pathology in several brain regions at 12 months of age. In a previous study, we have shown that overt motor symptoms in this mouse model are associated with elevated levels of α‐syn oligomers/protofibrils in the spinal cord from 12 months of age (Lindström, Fagerqvist, et al., 2014). The onset of pathology in this mouse model shows large interindividual variation, and we have observed some aggregated α‐syn in animals already from 4 months of age (Fagerqvist et al., 2013).

In this study, we explored whether (Thy‐1)‐h[A30P] α‐syn tg mice display symptoms at younger ages than 12 months and if such symptoms correlate to brain pathology or colonization of *H. pylori*. Early behavioral symptoms have not been previously shown in this mouse model and would be important readouts for treatment studies. Using a number of motor and behavioral tests, we could demonstrate fine motor impairments from 2 months of age and impaired locomotor behavior from 8 months of age in these mice. Moreover, a decreased tyrosine hydroxylase (TH) reactivity in the midbrain was found, which indicates a compromised dopaminergic function in the tg mice. However, neither levels of α‐syn nor those of inflammatory cytokines in the central nervous system correlated with any of the observed symptoms.

## 2 MATERIALS AND METHODS

### 2.1 Ethical considerations

All experiments involving mice were approved by the Uppsala Animal Ethics Committee. The use and care of the animals were conducted in accordance with the EU Directive 2010/63/EU for animal experiments.

### 2.2 Animals

An equal number of male and female homozygous (Thy‐1)‐h[A30P] α‐syn tg mice, expressing human α‐syn with the A30P‐mutation under the Thy‐1 promoter, were used (Kahle et al., 2000). The human [A30P] α‐syn transgene had been injected in hybrid B6/DBA oocytes, and founders were extensively (>10 generations) backcrossed into the C57BL/6J background (Frasier et al., 2005; Neumann et al., 2002). In total, 80 animals at 2, 8, and 11 months were included. As controls, we utilized age‐matched male and female wild‐type (wt) mice from the same background strain, C57BL/6J (born and housed in the same animal facility as the tg mice). For an overview, see Table 1. At 2 months of age, animals were only tested in the challenging beam test, while animals in the 8 and 11 months groups were tested in all three behavioral tests. All animals were housed in open cages, 1–5 animals per cage, on a 12:12 hr reversed dark: light cycle in a temperature‐ and humidity‐controlled room and were given food and water *ad libitum*. Gloves were used in all physical contacts with the animals.

**Table 1:** Number of animals at different ages (tg/wt controls) used for the challenging beam test, the hanging wire test, and the MCSF test

Behavioral test	2** **months	8** **months	11** **months
Challenging beam test	*n* = 12/*n* = 10	*n* = 16/*n* = 10	*n* = 19/*n* = 12
Hanging wire test	Not tested	*n* = 16/*n* = 10	*n* = 19/*n* = 12
MCSF test	Not tested	*n* = 17/*n* = 10	*n* = 19/*n* = 12

MCSF = multivariate concentric square field.

### 2.3 Behavioral testing

To reduce stress during testing, the investigator that performed each test also handled the animals on several occasions during the week prior to testing. On the test day, animals were transferred to the test room in their home cage and were allowed to adapt for a minimum of 40 min. The behavioral tests were performed during subsequent weeks (in order to allow animals to get rest between tests) in the following order; multivariate concentric square field™ test (MCSF™) test, hanging wire test, and challenging beam traversal test. All tests were performed during the dark phase of the light cycle with males and females tested separately. Body mass was measured before each cycle of behavioral testing and was controlled throughout the study. All animals included in the study were randomly given an identification number to ensure blinding during analysis of data.

### 2.4 Challenging beam traversal test

The challenging beam test was performed on 2‐, 8‐, and 11‐month‐old mice according to a previously published protocol (Fleming et al., 2004). This test has a continuous increase in difficulty, and the apparatus consists of a plexiglas beam divided into four 25‐cm sections; 35, 25, 15, and 5 mm wide. Two days of training were performed for habituation to the task. The first day included habituation to the goal house, and the second day included training on the beam without the grid. During testing, a mesh grid (1 cm^2^) of corresponding width was placed over the beam surface. Animals were videotaped while traversing the grid‐surfaced beam for a total of three trials. A blinded observer viewed videotapes, and the number of steps and frequency of errors was scored by watching the video in a slow‐motion mode. An error was defined as when a forelimb or hindlimb slipped through the grid and became visible between the grid and the beam surface or on the side of the grid during a forward movement. Errors per step scores and number of steps were calculated across all three trials.

### 2.5 Hanging wire test

The hanging wire test was performed on 8‐ and 11‐month‐old mice by placing the mouse on a wire (3 mm in diameter) and letting it hang in its forepaws at a height of 38 cm from the table surface. Soft bedding (paper sheets) was placed below the wire to prevent fall injuries. The total test time was set to 30 s, and the test was completed after that each animal had completed three trials. The mice were alternated in a given order to ensure that they had the same resting time between the trials. The wire was cleaned with ethanol between each mouse. The scale used to evaluate the hanging wire test is shown in Figure 2e. A combined score for the use of hind legs and tail when climbing on the wire (body score) and the time to either reach one of the columns or fall off the wire (time score) were summarized to a score with a maximum of 11. The time to hang on to the wire as well as the best total score over three trials was included for the statistical analyses. One of the 8‐month‐old α‐syn tg mice was removed from the analysis due to malformed paws.

### 2.6 Multivariate concentric square field™ test

In order to measure locomotor activity and obtain a behavioral profile of the mice, the MCSF test was used (Meyerson, Augustsson, Berg, & Roman, 2006) on 8‐ and 11‐month‐old mice. This test has previously been adopted to study functional outcomes and treatment effects after traumatic brain injury (Ekmark‐Lewen, Lewen, Meyerson, & Hillered, 2010; Ekmark‐Lewen et al., 2013, 2016). In the MCSF test, animals are placed in a novel arena, divided into different zones. Some of these zones are associated with a risk‐taking behavior (e.g., the elevated bridge or the open and unprotected center of the arena), whereas others are associated with safety‐seeking behavior (e.g., the dark corner room) or exploratory behavior (e.g., the hurdle zone with a hole board).

Mice were allowed to explore the arena for 20 min during which distance and velocity were assessed as a measure of general locomotor activity. Grooming behavior, number of rearing, fecal pellets, and presence of urine spots were also determined. Manual scoring of visited zones, rearing, and grooming behavior was performed using the software Score version 2.2 (Copyright Solids, Uppsala, Sweden). All trials were video‐recorded, and the Ethovision XT 11.0 software (Noldus Technology, Wageningen, Netherlands) was used to analyze the velocity and distance moved. One control mouse in the 8‐month‐old group and one tg mouse in the 11‐month‐old group were detected as outliers and were excluded from the principal component analyses.

### 2.7 Tissue sampling

Within 48 hr from the end of the last behavioral test, the 8‐ and 11‐month‐old mice were anesthetized with isoflurane before being transcardially perfused with 0.9% saline, followed by the removal of brain and spinal cord. The left brain hemispheres and the spinal cords were frozen on dry ice and the right brain hemispheres were fixed in 4% PFA, followed by 70% ethanol before the tissue was paraffin embedded.

### 2.8 Immunohistochemistry

The right hemispheres from *A30P* α*‐syn* tg mice and a corresponding number of age‐matched control mice were fixed in formaldehyde, embedded in paraffin, sagittally sectioned (5 μm), and mounted on glass slides. Two tissue sections, at the approximately level of 0.4 mm from the bregma, from each animal were analyzed for α‐syn, GFAP, and Mac‐2 (8 months *n* = 3, 11 months *n* = 3). For analysis of TH staining, two tissue sections from four different sagittal levels were selected (8 months *n* = 5, 11 months *n* = 5). The following primary antibodies were used: oligomer‐selective α‐syn antibody mAb38F (anti‐mouse, 0.4 μg/ml (Fagerqvist et al., 2013)), anti‐GFAP (anti‐rabbit, 5.8 μg/ml, Dako, Glostrup, Denmark), anti‐Mac‐2 (anti‐mouse, 4 μg/ml, Cedarlane Labs, Burlington, Ontario, Canada), and anti‐TH (anti‐rabbit, 1 μg/ml, Abcam, Cambridge, UK). Before staining with mAb38F, anti‐Mac‐2, and TH antibodies, antigen retrieval was performed by incubating the sections in a 25 mM citrate buffer (pH = 7.3) that was brought to boil and thereafter left to cool for 40 min at room temperature. For staining with the GFAP antibody, this step was substituted by a 5‐min proteinase K treatment (10 ng/ml in a solution of 50 mmol/L Tris‐HCl and 5 mmol/L EDTA, pH 8.0) at 37°C. Thereafter, all sections were treated with 70% formic acid for 5 min and endogenous peroxidases were blocked by treatment with DAKO REAL Peroxidase‐Blocking Solution, followed by permeabilization of the sections with 0.4% Triton‐X in PBS. All sections were incubated with the primary antibodies over night at 4°C. For mAb38F, a mouse‐on‐mouse kit (BMK‐2202, Vector Laboratories, Burlingame, CA) was used, while for the other antibodies sections were incubated with biotinylated secondary anti‐rat (Mac‐2) or anti‐rabbit (GFAP and TH) (1:250, Vector Laboratories) antibodies, followed by incubation with streptavidin conjugated to horseradish peroxidase (Mabtech). NovaRed substrate kit (SK‐4800, Vector Laboratories) was used for the development before the tissues were mounted with DPX (VWR, Stockholm, Sweden). Sections for TH analysis were counter‐stained with hematoxylin. As negative controls, sections were incubated with either primary or secondary antibody alone.

### 2.9 Quantification of tyrosine hydroxylase immunoreactivity

For quantification of TH immunoreactivity, the right hemisphere from 8‐ and 11‐month‐old A30P α‐syn tg mice (*n* = 4 per time point) and age‐matched wt controls (8 months, *n* = 2, 11 months, *n* = 3) were used. Two sections from four different levels were selected, at the levels of 0.48, 0.72, 0.84, and 0.96 (mm) from the bregma. Three regions of interest (ROI 1–3) corresponding to ventral tegmental area (VTA), substantia nigra (SN), and retrorubral field (RR) were selected for analysis. The images were taken at 20× magnification with a Nikon microscope (DMX1200F, Nikon Instruments Inc., Melville, NY) and converted to grayscale. Quantitative image analysis was performed with ImageJ software (NIH), and the percentage of area covered with pixels above threshold (area fractions) was obtained.

### 2.10 Proinflammatory cytokine measurement

The left hemispheres from 11‐month‐old *A30P* α*‐syn* tg mice (*n* = 19) and age‐matched wt control mice (*n* = 11) were selected for measurement of ten different cytokines using a multispot cytokine assay (V‐plex Proinflammatory Panel 1 (mouse), MSD). The brains were homogenized in PreCellys tubes (2 × 5 s at 4,500 g) in 1:5 (tissue weight: extraction volume ratio) in TBS buffer (137 mmol/L NaCl and 20 mmol/L Tris at pH 7.6) with complete protease inhibitor cocktail (Roche, Mannheim, Germany). To obtain 1:10 ratio, half of the homogenate was transferred into new LoBind Eppendorf tubes, before adding cold TBS and storing the remaining slurry. The homogenates were centrifuged at 16,000 g for 1 hr at 4°C, and the supernatant was stored in −70°C until analysis. Standards were made according to the manufacturer's protocol, and the samples were diluted twofold in diluent buffer. Duplicates of the standard and samples were added to the plate and incubated at room temperature for 2 hr with shaking, followed by incubation with SULFO‐Tag™‐labeled detection antibodies at room temperature for 2 hr with shaking. Read Buffer T was added, and the plates were analyzed with a MSD instrument (MSD).

### 2.11 Total and protofibrillar α‐syn ELISAs

For total and protofibrillar α‐syn analyses, the brain stem and spinal cord were chosen, as these areas have been previously found to display early α‐syn pathology (Fagerqvist et al., 2013). The tissue was homogenized in TBS (20 mmol/L Tris and 137 mmol/L NaCl, pH 7.6) with protease inhibitor (Roche, Mannheim, Germany) (1:10 tissue weight: extraction volume). Homogenization was followed by centrifugation at 16,000 g for 1 hr (4°C). The supernatant (TBS fraction) was saved and stored at −70°C until analyses. Total α‐syn levels were measured using a SECTOR Imager 2400 from Meso Scale Discovery (MSD, Meso Scale Diagnostics, LLC, Maryland). MSD plates (MULTI‐ARRAY^®^ 96‐Well Standard Plates) were coated with 0.5 μg/ml clone 42 α‐syn antibody (BD Biosciences, Franklin Lakes, NJ) in PBS and incubated over night at +4°C. Free binding sites were blocked by incubation with 1% Blocker A (MSD). After washing the plates 4× in TBS with 0.05% Tween‐20, samples and recombinant α‐syn standard (BioArctic, Stockholm, Sweden) were diluted in 1% Blocker A and added to the plates. Bound α‐syn was detected by a polyclonal rabbit anti α‐syn antibody (FL140, Santa Cruz Biotechnology, Santa Cruz, CA), followed by an anti‐rabbit antibody conjugated with a SULFO‐TAG (MSD). The plates were washed 4× in TBS with 0.05% Tween‐20 between each incubation step. After the last wash, 150 μl Read Buffer T (2×) (MSD) was added to each well and the light signal generated was measured in the SECTOR Imager. The α‐syn concentrations in the samples were quantified using the standard curve generated from recombinant α‐syn in the Discovery Workbench software (MSD).

Levels of α‐syn oligomers/protofibrils were measured using a sandwich ELISA, as previously described (Fagerqvist et al., 2013). In short, high binding 96‐well EIA/RIA plates (Corning Inc, Corning, NY) were coated with the oligomer‐selective antibody mAb38F in PBS overnight (4°C). The next day, plates were blocked with 1% BSA and incubated with the samples (TBS fractions from brain or spinal cord) before incubation with the detection antibody (biotinylated mAb38F) and streptavidin‐HRP (Mabtech AB, Stockholm, Sweden). K‐blue Aqueous TMB substrate (Neogen Corporation, Lansing, MI) was added to the plates, and the reaction was stopped with 1 M H_2_SO_4_ followed by analyses at 450 nm (Infinite M1000, Tecan, Männedorf, Switzerland).

### 2.12 Helicobacter analyses

In 8‐ and 11‐month‐old A30P α‐syn tg animals, fecal samples were collected from the colon after perfusion, stored on ice, and frozen at −20°C until analyses (Surrey Diagnostics, Cranleigh, UK). After confirmation of *Helicobacter* species in 16 of 35 tg animals using conventional PCR (Poynter, Phipps, Naranjo‐Pino, & Sanchez‐Morgado, 2009), positive samples were further analyzed for *Helicobacter pylori* (*H. pylori*) (Kawamata et al., 1996). Two samples were also analyzed in order to identify the unknown *Helicobacter* species using 16S sequencing primers for determination (GSK, UK).

### 2.13 Statistical analyses

For statistical analyses, Shapiro–Wilk's W test was used to analyze whether the data showed a normal distribution. Normally distributed data were analyzed with ANOVA followed by a parametric post hoc test. If data did not meet the assumption for normal distribution, the nonparametric Kruskal–Wallis analysis of variance of ranks was used for overall comparisons between groups, followed by Mann–Whitney *U* test for groupwise comparisons. The TH staining was analyzed using unpaired *t* test. Statistica 13.0 software (StatSoft Inc., Tulsa, OK) was used for statistical analyses, and a *p*‐value <.05 was considered statistically significant.

Principal component analysis (PCA) (Eriksson, Kettaneh‐Wold, Trygg, Wikström, & Wold, 2006) was used as a complement to traditional statistics as it provides an overview of the dataset and is a powerful tool to recognize patterns in data, such as outliers and groups. The analyses created a score plot showing a summary of the relationship among individuals and a loading plot in which variables important for these relationships can be identified. The two plots are complementary and superimposable. SIMCA‐P+ 14.0 software (Umetrics AB, Umeå, Sweden) was used for this purpose. All data are shown as mean ± *SD*.

## 3 RESULTS

### 3.1 Animals

**Figure 1:**
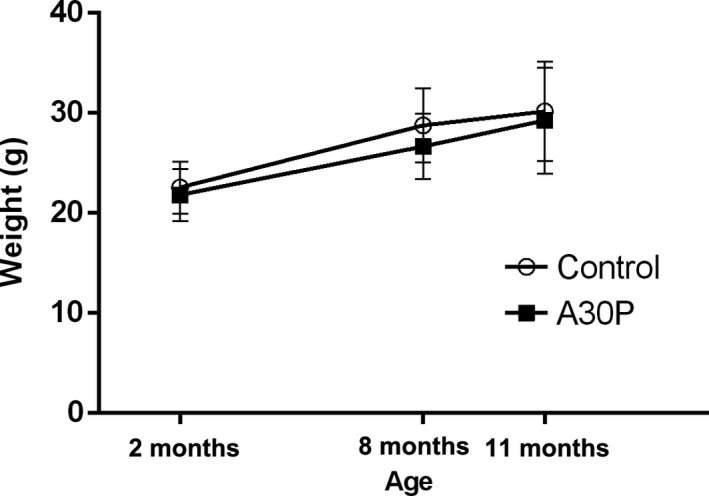
All mice were weighed before the first behavioral test at 2, 8, and 11 months of age. There was no difference in weights between A30P α‐syn tg mice and wt control mice in any of the age‐groups. Values are expressed as mean ± *SD*

There was no difference in body mass between A30P α‐syn tg and wt control mice for any of the age‐groups (Figure 1). The mice did not show any signs of overt motor symptoms or other behavioral deficits that could be detected in the home cage or during handling at any time point during the study.

### 3.2 Challenging beam traversal test

**Figure 2:**
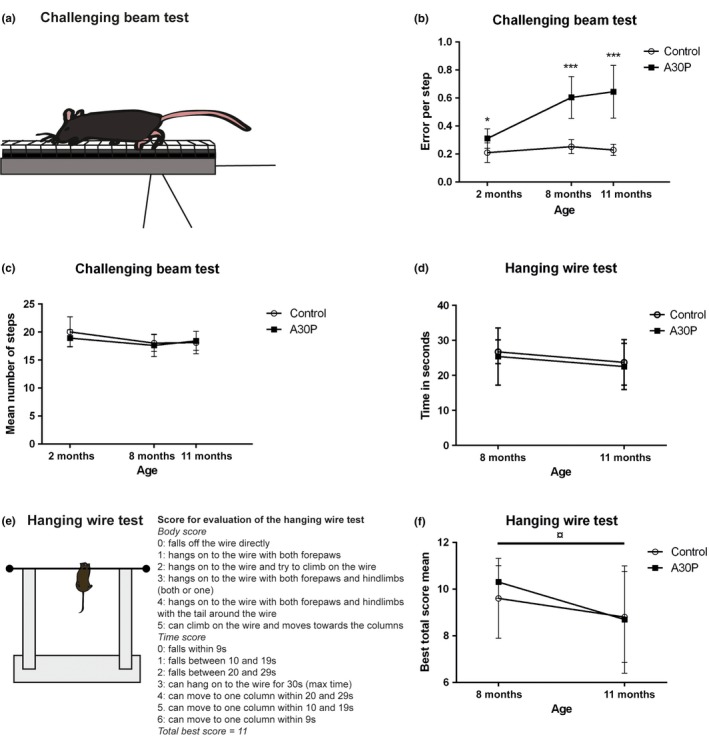
The challenging beam test for fine motor impairment consists of a 1‐m‐long and thinning beam with an overlaying mesh grid (a). The foot slips that the animals make while traversing the beam are counted. Animals are trained to traverse from the starting position at the thickest part of the beam to enter a goal house at the end of the thinnest part of the beam. The A30P α‐syn tg mice showed an increasing number of errors per step at 2, 8, and 11 months of age, compared to control animals (b), while no difference in the number of steps between groups could be detected (c). There was no difference in the time the mice managed to hang on to the wire, scored for three trials with a maximum time of 30 s (d). The hanging wire test was further evaluated using a combination of body score (0–5) and the time to fall or move to the column (0–6) with a total maximum score of 11. The best total score of three trials for each mouse was used for the statistical analyses (e). The A30P α‐syn tg mice did not differ in muscle strength compared to control mice at any of the investigated time points. The A30P α‐syn tg mice showed a significant worsening in performance between 8 and 11 months of age, but the same trend was seen for wt control animals (f). Values are expressed as mean ± *SD*, *p < .05, **p < .01, ***p < .001 compared to control animals

For the evaluation of fine motor performance, animals underwent the challenging beam test at 2, 8, and 11 months of age. At all investigated ages, the A30P α‐syn tg mice made more errors per step compared to the wt control mice (0.31 vs. 0.21 for 2‐month‐old mice, *p* = .011, *U* = 22.0; 0.6 vs. 0.25 for 8‐month‐old mice, *p* = .000035, U = 1.0; and 0.65 vs. 0.23 for 11‐month‐old mice, *p* = .000004, U = 0.0) (Figure 2b). The mean total number of steps (as defined by the number of times the right forepaw moves forward) made during three trials did not differ between the tg and control groups at any time point (Figure 2c).

### 3.3 Hanging wire test

The hanging wire test was used for measurement of muscle strength and coordination in 8‐ and 11‐month‐old mice according to the scale shown in Figure 2e. There were no differences between the A30P α‐syn tg mice and the wt control mice at any of the time points and there was a high variation within the groups, especially at 11 months (Figure 2f). A weakened performance was seen between 8 months of age compared to 11 months of age, for the A30P α‐syn tg mice (from total best score 10.3–8.8, *p* = .011, *U* = 74.5), while the wt control mice displayed a nonsignificant trend for a worsened performance over time.

### 3.4 Multivariate concentric square field™ test

**Figure 3:**
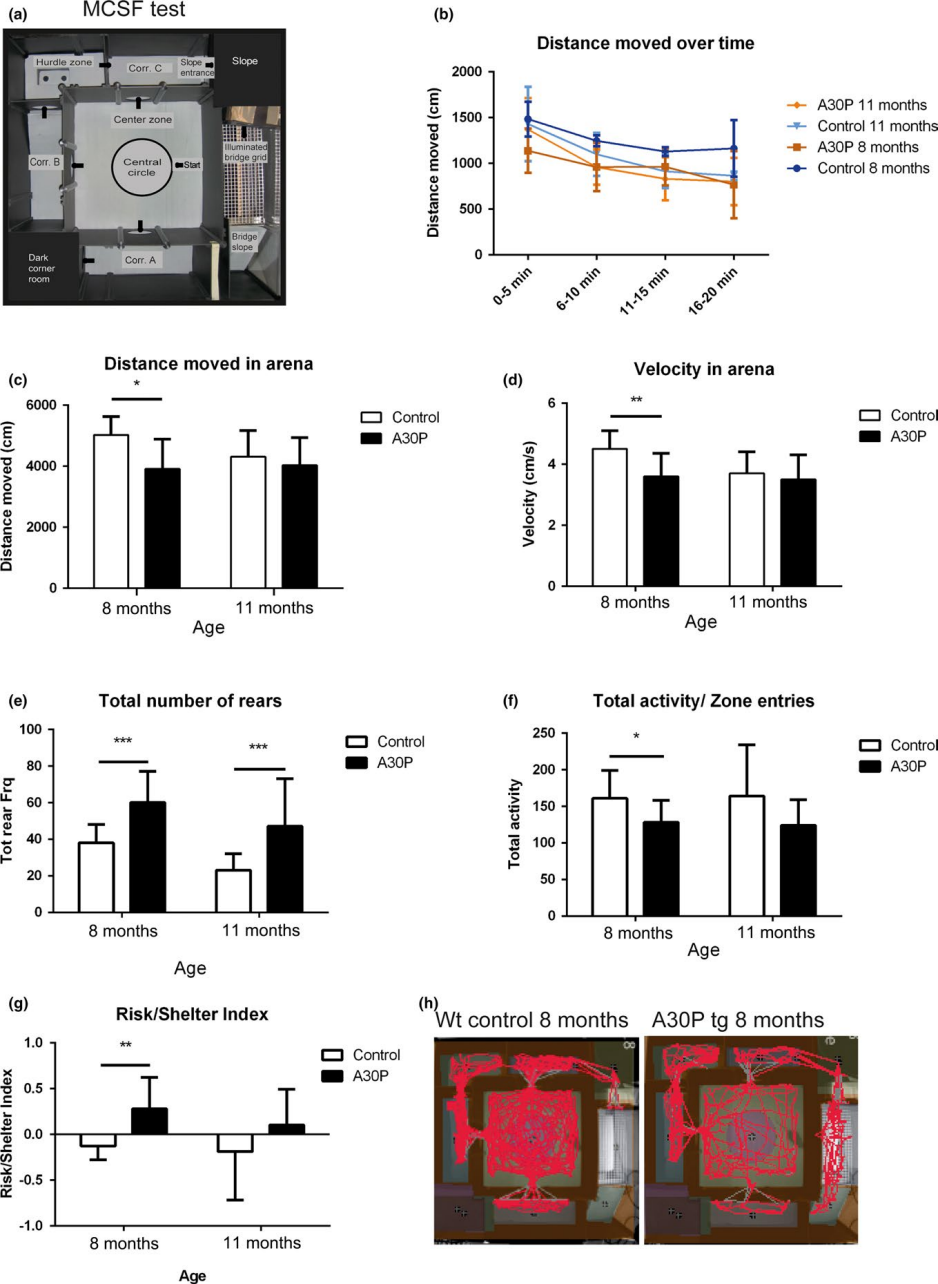
The multivariate concentric square field (MCSF) test arena with the defined zones labeled. Animals were placed in the center zone at the start position and were allowed to explore the arena for 20 min. Arrows show the different paths that the animals could take. Corr A–C = Corridors A–C (a). The distance moved in the MCSF arena decreased slightly over time, for all age‐groups regardless of genotype (b). The distance moved (cm) and the velocity (cm/s) in the MCSF arena were significantly lower in A30P α‐syn tg animals compared to control animals at 8 months (c, d). The number of rearings (wall rearing and free rearing combined) was increased in 8‐ and 11‐month‐old A30P α‐syn tg mice compared to wt age‐matched controls (e). The A30P α‐syn tg mice entered less zones compared to controls at 8 months of age (e). The risk/shelter index was increased in the A30P α‐syn tg mice as compared to control mice at 8 months of age (g). Representative images of tracks in the MCSF test show that the A30P α‐syn tg mice displayed a reduced general activity, but crossed the illuminated bridge area more compared to control animals (H). Values are expressed as mean ± *SD*, *p < .05, **p < .01, ***p < .001

The A30P α‐syn tg and wt control mice were evaluated with the MCSF test at 8 and 11 months of age. The MCSF test is divided into various zones, and many different behaviors can be measured during one test trial (Figure 3a). In total, 99 variables were recorded in the MCSF test, including duration, frequency and latency to the different zones, rearing, and grooming behavior, as well as urination and fecal pellets. The distance moved (in cm), velocity (cm/s), and the number of zone entries were assessed as a measurement of general locomotor activity. All groups of animals adapted to the novel environment and decreased their distance moved from the first 5 min to the last 5 min in the arena (Figure 3b). In general, the A30P α‐syn tg mice had a lower locomotion compared to wt mice. However, the activity degree of the tg mice did not differ between 8 and 11 months of age, while the wt control animals decreased their locomotion (shorter distance and lower velocity) from 8 to 11 months of age. Significant differences in the moved distance between A30P α‐syn tg and control mice were found at 8 months of age (3,909 cm compared to 5,021 cm, *p* = .003, *U* = 29.0) (Figure 3c,d). At 11 months of age, the control animals had decreased their activity in the arena toward the same degree as A30P α‐syn tg animals (4031 cm compared to 4306 cm). The A30P α‐syn tg animals showed an increase in rearing behavior compared to control mice, at both 8 months (65 times compared to 34 times, *p* = .00036, *U* = 13.5) and 11 months (54 times compared to 26 times, *p* = .0007, *U* = 30.0) of age (Figure 3e). Moreover, the A30P α‐syn tg mice entered less zones compared to controls at 8 months of age (128 compared 161, *p* *=* .027, *U* *=* 41.5). The same trend was seen for mice at 11 months of age, but the group differences did not reach the level of significance (Figure 3f). Moreover, an increased risk/shelter index was found for the A30P α‐syn tg mice compared to control mice at 8 months of age (0.28 compared to −0.13, *p* = .0023, *U* = 15.5) (Figure 3g), indicating that these mice have a higher risk‐taking behavior, based on visits to the illuminated bridge area. Representative images of exploration tracks in the MCSF test illustrate that tg mice were less active in the MCSF arena but visited the exposed bridge area more often compared to the control animals (Figure 3h).

**Figure 4:**
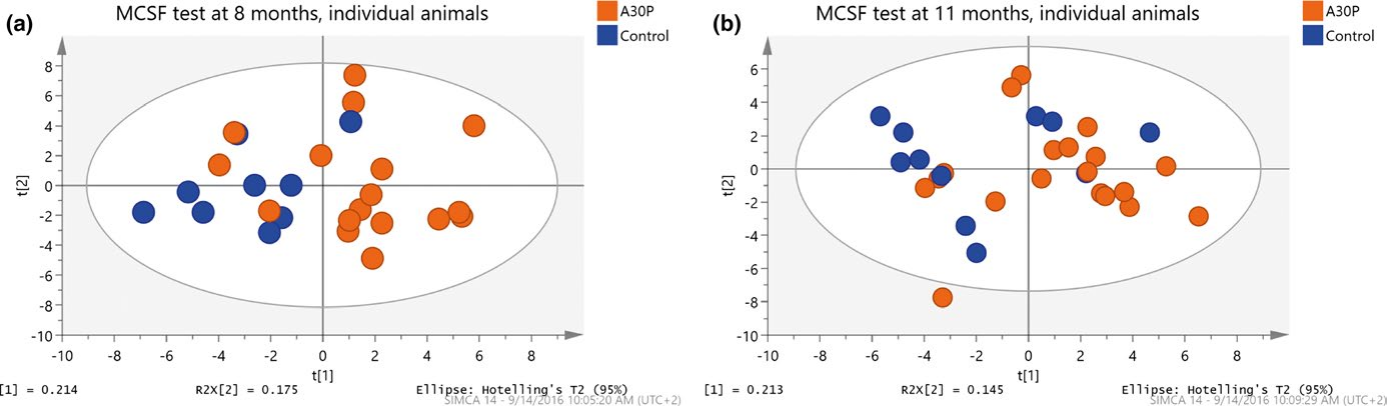
Principal component analysis, including all variables in the multivariate concentric square field (MCSF) test, displays the scores of the two‐first principal components (t_1_ and t_2_) for individual mice at 8 (a) and 11 (b) months of age. The A30P α‐syn tg mice (orange) were mainly positioned to the right of the score plots and were thus separated from age‐matched wt control mice (blue) that were mainly positioned in the left side of the score plot, based on their behavior in the MCSF arena

Based on the MCSF test analyses, the A30P α‐syn tg mice revealed a different behavioral profile than the control mice in the score plot from the PCA (Figure 4a,b). The PCA, including all variables in the MCSF test, displays the scores of the two first principal components (t1 and t2) for individual mice at 8 and 11 months of age. Most of the A30P α‐syn tg mice were located in the right part of the score plot while the control animals were located in the left part, based on their behavior in the MCSF arena. The following variables contributed most strongly to the separation of the A30P α‐syn tg and control mice: more visits to the bridge, higher risk/shelter index, and a lower activity degree shown by less distance moved in the arena with a lower velocity (Figure S1a,b). In summary, the A30P α‐syn tg mice had a lower general activity and were taking more risks, including making more entries to the illuminated bridge area, as compared to the control mice.

### 3.5 Immunohistochemical analyses

**Figure 5:**
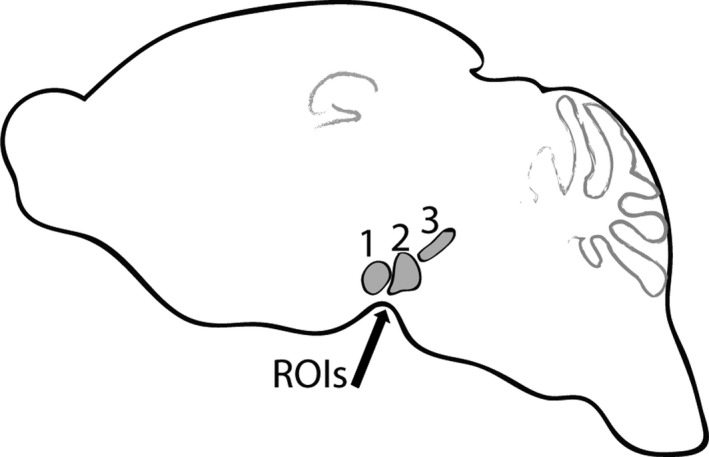
Regions of interest (ROI 1–3) included ventral tegmental area (ROI 1), substantia nigra (ROI 2), and retrorubral field (ROI 3). All three ROIs were used for quantification of TH immunoreactivity

The right hemispheres from A30P α‐syn tg and wt control mice were collected at 8 and 11 months of age and analyzed for α‐syn staining and inflammatory markers. For analysis of TH immunoreactivity, three regions of interest (ROIs), including the ventral tegmental area (VTA), substantia nigra (SN), and retrorubral field (RR), were chosen (Figure 5).

**Figure 6:**
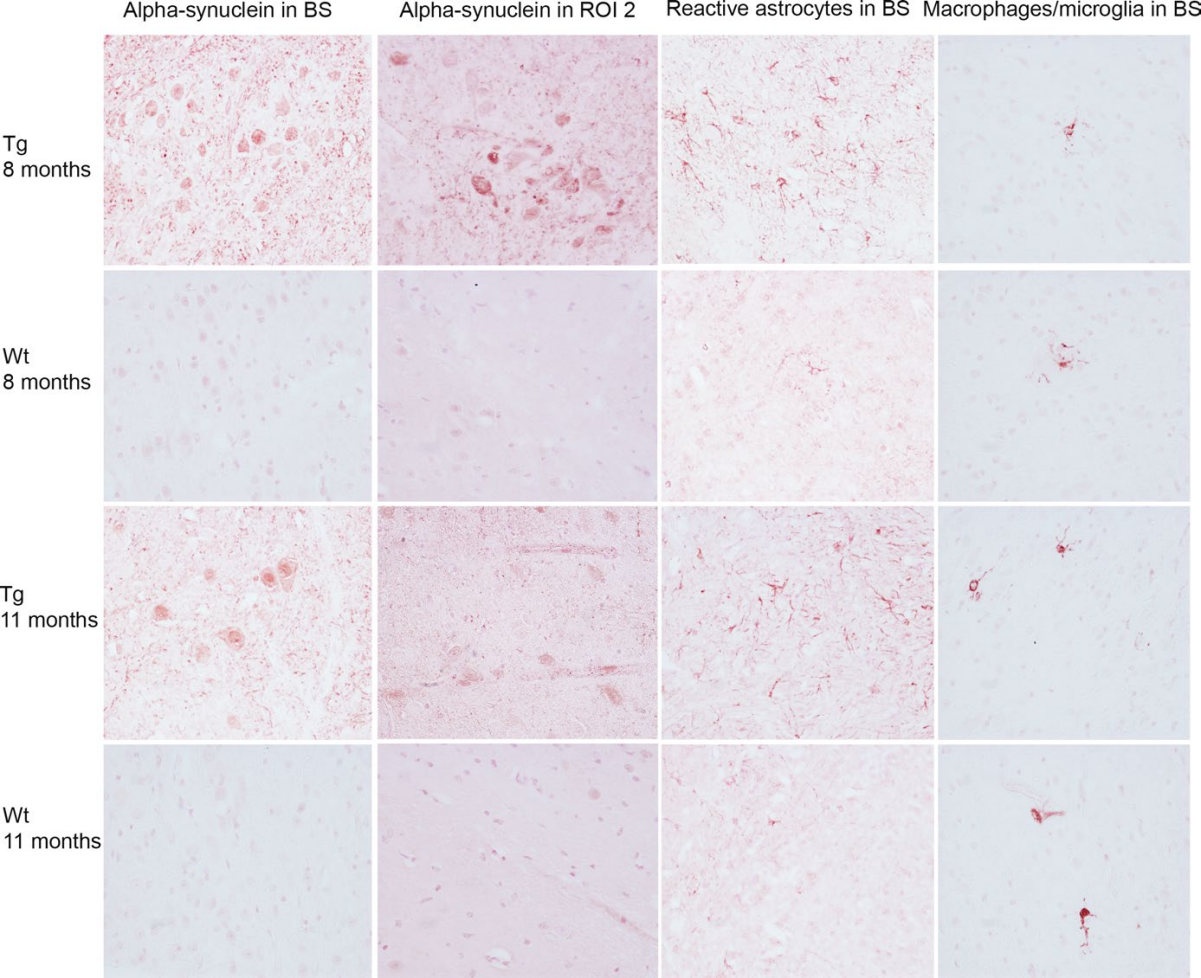
Immunohistochemical staining of α‐syn (mAb38F), astrocytes (GFAP), and activated macrophages/microglia (Mac‐2) in 8‐ (*n* = 3) and 11 (*n* = 3)‐month‐old tg and wt control mice. Representative images captured at 40× of the brainstem (BS) and region of interest (ROI) 2, which includes the substantia nigra. The A30P α‐syn tg mice displayed some aggregated α‐syn staining in the brain stem and in ROI 2. The A30P α‐syn tg mice displayed some increase in GFAP staining in the brain stem, compared to wt control mice, but no difference in Mac‐2 staining between tg and wt mice was observed and the inflammatory response was overall sparse

With the oligomer‐selective α‐syn antibody mAb38F, the A30P α‐syn tg mice displayed staining primarily in the brainstem, but also to some extent in the midbrain and hippocampus at 8 (*n* = 3) and 11 (*n* = 3) months of age. The control mice did not show any mAb38F immunoreactivity. There was some increase in GFAP immunoreactivity (reactive astrocytes) in areas with increased α‐syn staining, in 8‐ and 11‐month‐old A30P α‐syn tg mice. The inflammatory response was sparse in 8‐ and 11‐month‐old A30P α‐syn tg mice and control mice, as indicated by the fact that only a few cells were positive forMac‐2, a marker for activated macrophages/microglia (Figure 6).

**Figure 7:**
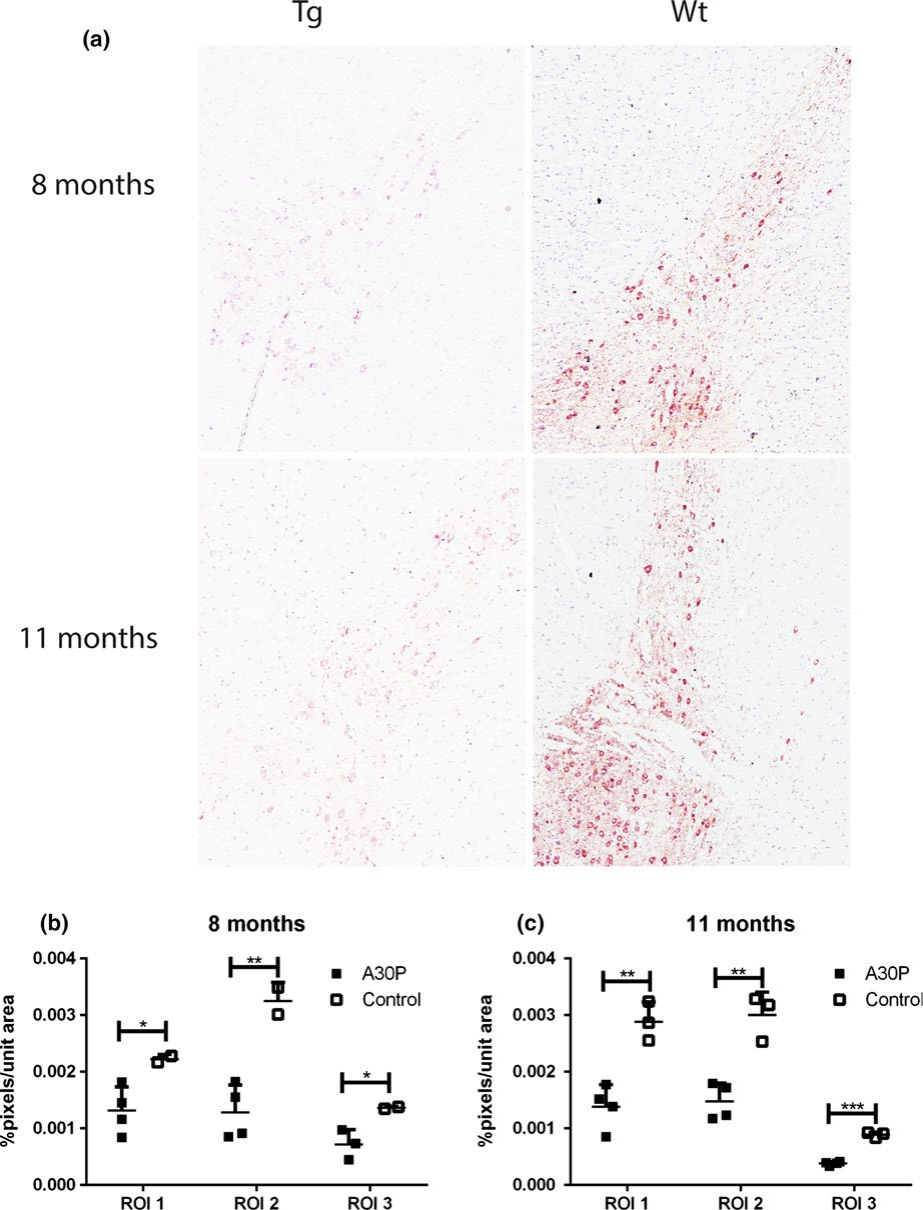
Representative images captured at 10×, including ROI 1–3, showed that A30P α‐syn tg mice displayed less TH immunostaining at both 8 and 11 months of age compared to wt control mice (a). Quantification of TH immunoreactivity showed that A30P α‐syn tg mice had a significant reduction in all areas compared to control mice. Moreover, these differences were more pronounced at 11 compared to 8 months of age (b, c). Values are expressed as mean ± *SD*, *p < .05, **p < .01, ***p < .001 compared to wt control animals

In order to analyze the dopaminergic system, immunoreactivity of TH in 8‐ and 11‐month‐old animals was quantified in three regions of interest (ROI 1–3), as shown in Figure 7. Representative images, including all ROIs, show that tg mice displayed significantly less TH immunoreactivity compared to wt control mice, at both 8 and 11 months of age (Figure 7a). Quantification of ROIs from two sections per animal at four different bregma levels verified a significant loss of TH immunoreactivity in 8‐ and 11‐month‐old A30P α‐syn tg mice compared to wt controls (Figure 7b,c).

### 3.6 Analyses of proinflammatory cytokine levels

V‐plex multiassay was used to measure ten proinflammatory cytokines (interleukin (IL)‐6, IL‐1β, IL‐2, IL‐4, IL‐10, IL‐12p70, IL‐5, KC/GRO (also known as neutrophil‐activating protein 3), interferon‐γ, and tumor necrosis factor‐α in the brain of 11‐month‐old A30P α‐syn tg and age‐matched control mice. The two groups of mice did not differ in terms of cytokine levels (Figure S2). Moreover, for the majority of cytokines, the levels were just above the lower limit of detection.

### 3.7 Assessment of α‐synuclein levels

**Figure 8:**
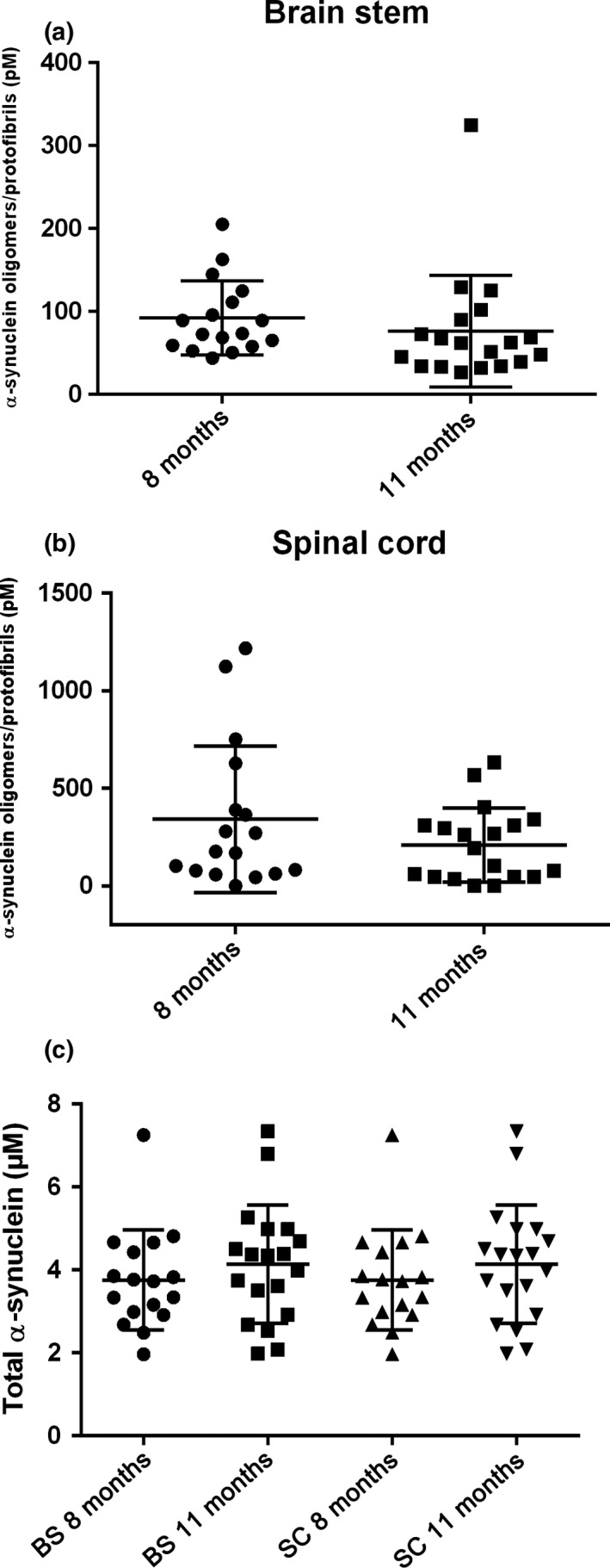
Total and oligomeric/protofibrillar α‐syn levels in the spinal cord and brain stem of A30P α‐syn tg mice. Levels of α‐syn oligomers/protofibrils (a, b) and total α‐syn (c) in the spinal cord (SC) and brain stem (BS) of A30P α‐syn tg mice at 8 months (*n* = 17) and 11 months (*n* = 19)

The levels of total α‐syn and α‐syn oligomers/protofibrils in spinal cord and brain stem were analyzed by ELISA in 8‐ and 11‐month‐old mice. The levels were under the detection limit in both regions of the wt control mice, while the A30P α‐syn tg animals displayed a large variability, ranging from levels under the detection limit to about 1,200 pmol/L in spinal cord and 100 pmol/L in brain stem (Figure 8a,b). There was no difference in levels of total α‐syn between 8 and 11 months of age in neither of the two regions (Figure 8c). The levels of α‐syn (total and oligomeric/protofibrillar) did not correlate with errors in the challenging beam test nor with the outcome in the MCSF test.

### 3.8 *Helicobacter* species

In order to investigate the presence of *helicobacter* species in A30P α‐syn tg mice, fecal pellets were collected from the colon of 8‐ and 11‐month‐old A30P tg mice at the time when the mice were sacrificed. In the 8‐ and 11‐month‐old A30P α‐syn tg mice, 16 of 35 animals showed positive results for *Helicobacter* species, but none of the animals were positive for *H. pylori*. The *Helicobacter* species found showed close similarity with *H. pylori* and further analysis showed a positive result for *H. typhlonius* and *H. apodemu*s, both *H. pylori*‐related branches. However, no correlations between the presence of these species and higher α‐syn levels or worse behavioral outcome were observed for any of the age‐groups.

## 4 DISCUSSION

Characterizing the phenotype in mouse models of α‐synucleinopathies can give new insights into the underlying disease mechanisms and provide us with important tools to assess effects of novel therapeutic strategies. For this purpose, it is critical to choose tests that are sensitive enough to allow detection also of subtle symptoms in these models. As for the (Thy‐1)‐h[A30P] α‐syn tg mice, no such early behavioral or motor features have been reported. The mice display deposition of phosphorylated α‐syn from 8 to 9 months of age, predominantly in the brain stem and spinal cord (Neumann et al., 2002). In addition, they have been shown to suffer from behavioral disturbances from 12 months of age (Freichel et al., 2007). Moreover, a progressive deterioration of locomotor function, including an unsteady gait and a weakening of the extremities as well as an impaired locomotor performance, was reported from this age. However, earlier behavioral or motor anomalies in these mice have not previously been reported, although we were able to identify aggregated α‐syn in the upper part of the brain stem already at 4 months of age (Fagerqvist et al., 2013).

The aim of this study was to investigate whether it would be possible to detect fine motor and behavioral impairments that would correlate to earlier stages of protein aggregation in the (Thy‐1)‐h[A30P] α‐syn tg mice. Fine locomotion and gait deficits, muscle strength, and behavioral profiles were analyzed in tg and age‐matched wt control mice at 2, 8, and 11 months of age, i.e. at time points that precede the development of more overt motor symptoms and widespread pathology in these mice. Mice at 8 and 11 months of age were subjected to the challenging beam test, the hanging wire test, and the MCSF test, while the 2‐month‐old mice were investigated with the challenging beam test only.

Fine motor impairments, as assessed by the challenging beam test, were detected already in 2‐month‐old A30P α‐syn tg mice, and these symptoms progressed up to 11 months of age. In the hanging wire test, the A30P α‐syn tg mice had an unaffected muscle strength and coordination compared to wt control animals, in concurrence with what has previously been reported for these animals (Freichel et al., 2007). There was no group difference regarding the time that the mice managed to hang on to the wire and most mice, both tg and controls, were climbing on the wire toward one of the columns instead of just hanging on to it. In order to measure motor coordination, we scored how well animals used their hind legs and tail when climbing on the wire. Over time, there was a slight decrease in the hanging wire performance in both groups, based on the combined score for time on the wire and motor coordination, but the age‐related worsening in performance was significant only in the tg group. Moreover, a large intergroup variation could be seen for this test, and for future studies, this test could be modified by instead using wires of varying thicknesses and allow a longer maximal time for testing.

Behavioral profiling with the MCSF test showed that the A30P α‐syn tg mice had an impaired locomotor function, where they moved a shorter distance at a lower velocity as compared to control mice. The A30P α‐syn tg mice also made less visits to different zones in the MCSF test arena, indicating an impairment in exploratory activity. At the same time, these animals showed an increased rearing behavior. Spontaneous rearing behavior is a natural response to a novel environment in many mammals, including mice. There are many factors that may affect rearing, including responses to stress, anxiety and fear, hippocampal lesions, and different pharmacological treatments (reviewed in (Lever, Burton, & O'Keefe, 2006)). There are contradictory reports as to whether and, if so, how locomotor and rearing behaviors are correlated with each other. A previous report has shown that locomotor activity is positively correlated with rearing activity in the open field test (van Abeelen, 1977). However, another study failed to show such a correlation and instead found that frequently rearing mice had decreased locomotion compared to mice that were rearing less frequently (Crusio, Schwegler, Brust, & Van Abeelen, 1989). Similarly, the A30P α‐syn tg mice were rearing more frequently with decreased locomotor activity, as compared to control animals. The underlying reasons for the relationship between rearing and locomotor activity in these mice are unknown and warrant further investigation. Compared to the open field test, the MCSF test encourages exploration and has a more complex outline. By leaving the center zone of the test arena, animals can visit different zones and the motivation to explore can be measured. The A30P α‐syn tg mice left the center zone in order to visit other zones as fast as control animals, indicating that motivation and exploratory drive are not affected in these animals. Interestingly, the A30P α‐syn tg mice made more and longer visits to the bridge area as compared to the safer dark corner room, thus showing an increased risk/shelter index. At 11 months of age, the A30P α‐syn tg mice showed a pronounced impairment in fine motor function compared to control animals.

Staining with an α‐syn oligomer‐selective antibody displayed accumulated α‐syn, primarily in the brainstem, with some increase in GFAP immunoreactivity in the same area. These observations confirm our and others previous findings that the inflammatory response is sparse and confined to regions with α‐syn pathology (Lindström, Ihse, et al., 2014; Neumann et al., 2002). Similar to control mice, there were only few activated microglia/macrophages in the brainstem of tg mice, with no obvious correlation with regions with increased pathology. In line with the limited inflammatory staining, measurements of ten different proinflammatory cytokines did not show any differences compared to controls. However, most aggregated α‐syn could be observed in the brain stem but the cytokines were measured in the whole left hemisphere. It can therefore not be ruled out that differences might have been observed if the cytokines would have been measured in the brain stem only. Taken together, these results confirm that there is limited activation of the inflammatory response in young α‐syn tg mice.

As measured by the MCSF test, various parameters, such as distance moved and velocity, showed significant differences between A30P α‐syn tg and control mice at 8 months of age that were no longer seen at 11 months. Apparently, control animals decreased their locomotor behavior as a part of the aging process, while the tg mice did not change their performance at this later time point. The onset of pathology and overt motor symptoms is highly variable in this α‐syn tg mouse model (Freichel et al., 2007), which can explain why some mice at 8 months of age displayed a more evident behavioral phenotype compared to some of the 11‐month‐old mice. Moreover, this heterogeneity may also explain why there was no difference in pathological α‐syn staining or in levels of α‐syn oligomers between 8‐ and 11‐month‐old mice.

Previous studies have implicated that α‐syn can affect synthesis, metabolism, and release of dopamine (Kurz et al., 2010; Nemani et al., 2010; Perez et al., 2002). Moreover, a correlation between decreased TH immunoreactivity, α‐syn accumulation, and neuronal loss has been observed in the PD brain (Mori et al., 2006). Such a reduction in TH activity could possibly be seen as a neuroprotective mechanism, as decreased dopamine synthesis reduces cytotoxic α‐syn oligomers (Sidhu, Wersinger, & Vernier, 2004). In another transgenic α‐syn mouse model, a reduction in TH‐positive nerve terminals within the striatum but not in the SN was observed (Masliah et al., 2000).

As for the (Thy‐1)‐h[A30P] α‐syn tg mice, it has previously been reported that they do not differ from control mice in terms of dopamine levels in the striatum and frontal cortex at 8 months of age (Neumann et al., 2002). Here, we found that this mouse model indeed displays a reduced TH immunoreactivity in ventral tegmental area, substantia nigra, and retrorubral field, at both 8 and 11 months of age, compared to wt mice. Interestingly, we could also observe accumulated α‐syn in the same regions at the same time points, which may explain some of the movement impairments observed in the mice. The correlation between TH levels and more subtle motor impairments needs to be further investigated in younger animals—as it remains open whether also other mechanisms than loss of dopaminergic neurons may explain the earliest motor deficits in these mice. For example, mice overexpressing wt human α‐syn under the Thy1 promoter (Thy1‐aSyn) were found to have mild sensorimotor impairments at 2–8 months of age (Fleming et al., 2004, 2006), while the symptoms were neither L‐dopa responsive nor related to nigrostriatal cell loss. Thus, these mice may model a prodromal (i.e., “premanifest”) phase of PD. In addition, some of the α‐syn tg mice were described to have olfactory impairments and colonic dysfunction (Fleming et al., 2008). Such mice displayed high levels of α‐syn and proteinase K‐resistant inclusions containing α‐syn in various brain regions, including SN and locus coeruleus (Fernagut et al., 2007; Rockenstein et al., 2002).

We have previously seen a correlation between elevated α‐syn oligomer/protofibril levels in the spinal cord and severe motor symptoms in aged (from 12 to 17 months of age) A30P α‐syn tg mice (Lindström, Fagerqvist, et al., 2014). In the current study, the α‐syn tg mice displayed no differences in total α‐syn levels in spinal cord and brain stem between 8 and 11 months of age. Moreover, the levels of α‐syn oligomer in the spinal cord and brain stem were low and showed a high interindividual variability. We have previously observed that this α‐syn tg mouse model develops pathology at various time points and that the levels of α‐syn oligomers/protofibrils display a large interindividual variation (Fagerqvist et al., 2013). As expected at this early age, when behavioral and motor disturbances only can be measured with sensitive tests, there was no correlation between oligomer/protofibril levels and behavioral outcome.

The increased prevalence of *H. pylori* infection in patients with PD (Altschuler, 1996; Dobbs, Dobbs, Weller, & Charlett, 2000) suggests a link between *H. pylori* infection and idiopathic PD. Moreover, protein aggregation can be initiated through *H. pylori* infection (reviewed in (Cover & Blanke, 2005). We thus hypothesized that the presence of this bacterium could promote aggregation of α‐syn, resulting in higher levels of oligomers/protofibrils in infected mice and explaining the variation in levels of α‐syn oligomers/protofibrils in these mice. Analyses of *Helicobacter* species in the α‐syn tg mice indeed showed that half of the animals in the investigated age‐groups were infected with the *H. pylori*‐related forms, *H. typhlonius* and *H. apodemus,* that are also known to cause gastrointestinal tract infections in mice. However, we were not able to find any correlation between colonization of such *Helicobacter* species and motor/behavioral symptoms or pathological features in these mice.

## 5 CONCLUSIONS

We can here demonstrate that the (Thy‐1)‐h[A30P] α‐syn tg mice already at 2 months of age develop fine motor symptoms that progress with increasing age, thus preceding the manifestation of overt motor symptoms and widespread pathology in this mouse model. Moreover, at 8 months, tg mice showed a decreased general activity with increased risk‐taking behavior in the MCSF test for behavioral profiling. Thus, our results strongly indicate that the (Thy‐1)‐h[A30P] α‐syn tg mouse is a useful model to study more subtle symptoms and effects of early pharmacological intervention for disorders with α‐syn pathology.

## CONFLICT OF INTEREST

None declared.
