# Supplementary figures and images for: Early fine motor impairment and behavioral dysfunction in (Thy‐1)‐h[A30P] alpha‐synuclein mice
Source: Brain Behav. 2018 Feb 4;8(3):e00915. doi: 10.1002/brb3.915 (PMC5840441; doi:10.1002/brb3.915)

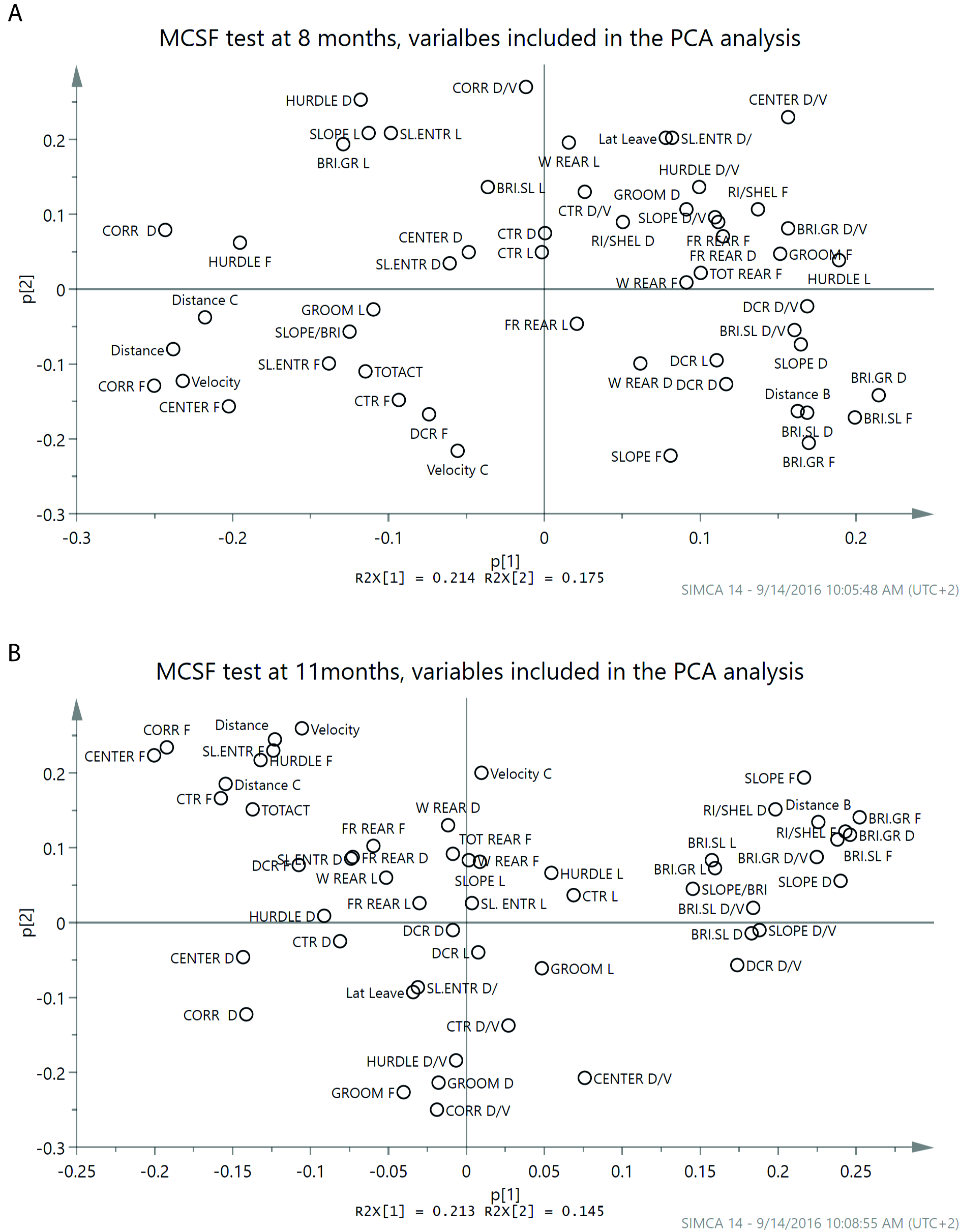

Supplement: Supplementary file 1 [file BRB3-8-e00915-s001.tif]
