# Supplementary material for: Early fine motor impairment and behavioral dysfunction in (Thy‐1)‐h[A30P] alpha‐synuclein mice
Source: Brain Behav. 2018 Feb 4;8(3):e00915. doi: 10.1002/brb3.915 (PMC5840441; doi:10.1002/brb3.915)
Supplement: Supplementary file 3 [file BRB3-8-e00915-s003.docx]

***Supplementary Figure 1*.** The loading plots displays the loading vectors (p_1_ and p_2_) illustrating the relationship between the variables included in the PCA analysis. Variables that are located further away from the origin are most important for the model (A, B). BR. GRI, bridge grid; C, center; CORR, corridors; D, duration; DCR, dark corner room; D/V, duration/visit; F, frequency; FR, free; L, latency; RI/SHEL, risk/shelter index; SL, slope; SL. ENTR, slope entrance; TOTACT, total activity.

***Supplementary Figure 2.*** The left hemisphere from 11 mo old A30P tg and control mice were used for cytokine measurement of ten different cytokines; interleukin (IL)-6, IL-1β, IL-2, IL-4, IL-10, IL-12p70, IL-5, KC/GRO: also known as neutrophil-activating protein 3, interferon (IFN)-γ and tumor necrosis factor (TNF)-α. The levels of all ten measured cytokines were overall low (in several animals under detection limit) and no significant difference in fold change, based on the lower level of detection, was seen between A30P tg and control mice at this time-point. Values are expressed as mean ± SD. For IL-12p70 controls and TNF-α controls and A30P tg there were only one animal that expressed values over the lower limit of detection, explaining why no standard bar is shown
